# Chronic Subordination Stress Induces Hyperphagia and Disrupts Eating Behavior in Mice Modeling Binge-Eating-Like Disorder

**DOI:** 10.3389/fnut.2014.00030

**Published:** 2015-01-06

**Authors:** Maria Razzoli, Valentina Sanghez, Alessandro Bartolomucci

**Affiliations:** ^1^Department of Integrative Biology and Physiology, University of Minnesota, Minneapolis, MN, USA; ^2^Department of Neuroscience, University of Parma, Parma, Italy

**Keywords:** obesity, pair feeding, meal-pattern analysis, ghrelin, glucose

## Abstract

**Background:** Eating disorders are associated with physical morbidity and appear to have causal factors like stressful life events and negative affect. Binge-eating disorder (BED) is characterized by eating in a discrete period of time a larger than normal amount of food, a sense of lack of control over eating, and marked distress. There are still unmet needs for the identification of mechanisms regulating excessive eating, which is in part due to the lack of appropriate animal models. We developed a naturalistic murine model of subordination stress-induced hyperphagia associated with the development of obesity. Here, we tested the hypotheses that the eating responses of subordinate mice recapitulate the BED and that limiting hyperphagia could prevent stress-associated metabolic changes.

**Methods:** Adult male mice were exposed to a model of chronic subordination stress (CSS) associated with the automated acquisition of food intake and we performed a detailed meal pattern analysis. Additionally, using a pair-feeding protocol we tested the hypothesis that the manifestation of obesity and the metabolic syndrome could be prevented by limiting hyperphagia.

**Results:** The architecture of feeding of subordinate mice was disrupted during the stress protocol due to disproportionate amount of food ingested at higher rate and with shorter satiety ratio than control mice. Subordinate mice hyperphagia was further exacerbated in response to either hunger or to the acute application of a social defeat. Notably, the obese phenotype but not the fasting hyperglycemia of subordinate mice was abrogated by preventing hyperphagia in a pair-feeding paradigm.

**Conclusion:** Overall, these results support the validity of our CSS to model BED allowing for the determination of the underlying molecular mechanisms and the generation of testable predictions for innovative therapies, based on the understanding of the regulation and the control of food intake.

## Introduction

A substantial number of obese individuals report binge-eating episodes, psychiatric conditions, stressful life events, more medical complaints, and a poorer quality of life (1–4). At the same time, stress and negative affect are increasingly recognized as risk factors for binge-eating and obesity (4–7). The parallel increase in incidence of seemingly independent psychiatric and metabolic diseases has long been suspected to be due to common causal factors.

Binge eating is currently included as an eating disorder in the Diagnostic and Statistical Manual of Mental Disorders [DSM-V, 5th ed.; (2)]. Binge-eating disorder (BED) is characterized by recurrent episodes of ingestion of large amount of food in discrete periods of time accompanied by feelings of loss of control and marked distress in the absence of compensatory behaviors such as purging, fasting, or excessive exercise. The difficulty to identify causal factors and underlying molecular mechanisms in humans makes animal models very valuable and needed to address mechanistic questions.

Unfortunately, current animal models do not recapitulate key traits of binging in humans and so far none matches the diagnostic symptoms of binging associated with obesity. Available animal models of binging are often based on a combination of acute stress exposure plus repeated cycles of food deprivation (8–10). Strikingly, most binge eating in humans is not driven by hunger or metabolic demands and most patients are overweight or obese [5th ed.; DSM–V; (2, 8, 11)]. At the same time, a significant number of individuals with BEDs report the onset of binges prior to dieting (12). Experimental animals from current models do not show significant increases in body weight/adiposity (13, 14), while the rebound hyperphagic response after fasting provides evidence that they are in an energy-deficient state (8, 9).

Over the years, we developed a naturalistic mouse model of chronic subordination stress (CSS) by which subordinate animals develop a complex behavioral and metabolic syndrome characterized by up-regulated hypothalamus–pituitary–adrenocortical axis functioning (increased plasma corticosterone, impaired negative feedback in the dexamethasone suppression test, down-regulated hippocampal glucocorticoids receptors) (15, 16), behavioral depression-like disorders (depression of activity, anhedonia, social withdrawal) (15, 16) as well as autonomic and immune-endocrine changes (15, 16). Importantly, hyperphagia arises spontaneously in subordinate mice and under a high-fat diet it is associated with vulnerability to obesity, metabolic-like, and type-2 diabetes-like syndromes (17–22). Arguably, this hyperphagic response represents the most robust phenotype in subordinate mice. Conversely, dominant animals do not manifest anxiety or depression-like behavior (17, 19) and develop a marked negative energy balance whereby hyperphagia is a mechanism to compensate for increased energy demands [(18, 21, 23); unpublished]. Based on previous data, here we set out to test the hypothesis that hyperphagia in subordinate animals has key features of binge-eating-like disorders. Furthermore, we also tested the hypothesis that selectively preventing hyperphagia with a pair-feeding protocol will be sufficient to limit the development of diet-induced obesity (21).

## Materials and Methods

### Animals

Ten- to twelve-week-old male Swiss CD1 mice (*n* = 69) were purchased from Charles River Laboratories and 10–12 weeks old male C57Bl6/J mice (*n* = 112) were purchased from Jackson Laboratories. All mice were maintained in a 12:12 hour light:dark cycle (lights on at 05:00 hours) at 22 ± 2°C. Mice were fed a standard diet (2018 Tecklad, Harlan; 3.1 kcal/g, 18% kcal from fat) unless otherwise specified. Animals were maintained and cared for in accordance with the NIH Guide for the Care and Use of Laboratory Animals. Experimental procedures were approved by the University of Minnesota Animal Care and Use Committee. The pair-feeding experiment was conducted at the University of Parma Italy using the same experimental conditions and experiments approved by local ethical committees of University of Parma and approved by the Italian Ministry of Health.

### Mouse model of chronic subordination stress

The protocol used represents a modified version of our previously published procedure (18, 20, 24). The experiment consisted of a 1-week pre-stress baseline phase and 4 weeks of CSS phase. During baseline, subjects were individually housed. During the 4 weeks following baseline, each CD1-resident mouse received an unfamiliar C57BL6/J or CD1 intruder mouse and the two animals were allowed to freely interact daily for 10 min, between 08:30 a.m. and 09:30 a.m. The same dyad remained paired for the total duration of the study. After the interaction leading to the social defeat of the C57BL6/J and one of the two CD1 mice, the two animals were separated by means of a wire-mesh partition thus allowing continuous sensory contact but no physical interaction. During the social interaction, offensive behaviors of the animals were manually recorded and mice social status was determined as previously established and detailed (17, 24). During the social interaction, offensive behaviors of the animals were manually recorded and subordinate mice social status was defined by the display of upright posture, flight behavior, and squeaking vocalization. Only dyads that reliably showed a stable dominant/subordinate hierarchy and in which the subordinate showed no attack after the fourth day of interaction were included in the study. Subordinate mice of both CD1 and C57BL6/J strains represented the experimental subjects in this series of experiments. Controls were represented by age, strain, and sex matched male mice housed in groups of three siblings that based on previous observations show no sign (immuno-endocrine and behavioral) of stress [see Ref. (24, 25) for further details]. Control animals were housed in group of 3 (from pre-existing groups of four to five animals per cage) the same day in which the chronic psychosocial stress procedure started and were received all the experimental manipulations as the experimental subjects except those involving the subordination stress. To conduct a detailed meal pattern analysis, individually housed mice were used as controls in specific experiments described below. Body weight gain was monitored weekly. Food intake was measured every other day (or as otherwise specified) during the baseline and stress phases. Food intake for group housed mice was divided for the number of animals in the cage.

### Meal-pattern analysis during chronic subordination stress

The BioDAQ episodic Food Intake Monitor for mice (BioDAQ, Research Diets, Inc., New Brunswick, NJ, USA) was used to investigate the microstructure of feeding. This made possible the continuous monitoring of meal pattern in the mouse housing-cage on a subset of control and subordinate subjects. During baseline, mice were individually housed and habituated to feeding through a low spill food hopper placed on an electronic balance mounted on the animal home cage. During the stress phase, the BioDAQ sensors and hoppers were fitted to the side of the CD1 resident cage where the C57BL6/J subject mouse was to be housed to continue the continuous monitoring of meal pattern with the exception of the daily social interaction, when the recording of feeding behavior was interrupted. In order to determine an accurate meal pattern analysis, control mice were maintained in individual home-cages fitted with the food hopper and the BioDAQ sensor as during baseline. The BioDAQ system records feeding bouts (changes in stable weight before and after a bout) as feeding bout vectors with a start time, duration, and amount consumed. Bouts are separated by an inter-bout interval, and meals consist of one or more bouts separated by an inter-meal interval. Meal pattern parameters were defined according to previously published literature (26–28). Meals were defined as feeding bouts occurring within 5 min of the previous response and with their sum equal to or greater than 0.02 g. If bouts of feeding were >5 min apart, they were considered as a new meal. Meal parameters assessed included meal frequency (number/period), meal size (grams/meal), meal duration (seconds/meal), total meal time (seconds/period), post-meal interval (PMI in seconds), eating rate (milligrams/minute), and the satiety ratio [calculated as the average PMI divided by the average meal size (minutes/gram food eaten)]. Parameters were calculated by the software provided by the manufacturer (BioDAQ Monitoring Software 2.2.02).

### Acute stress- and hunger-induced binge eating

The impact of CSS on the acute immediate consequences of a single social defeat on eating was determined measuring food intake during 6 h following social defeat on the first and 14th day of the stress phase.

Hunger-induced hyperphagia was monitored across 24 h following overnight fasting. Mice were not defeated on the testing day.

### Pair-feeding protocol during chronic subordination stress

To limit spontaneous hyperphagia and investigate its mechanistic role in stress-induced obesity, we used a pair-feeding protocol [i.e., preventing hyperphagia by pair-feeding mice to normal food intake; (29)]. CD1 mice were exposed to standard diet during baseline and the first week of chronic stress to establish dominance. High-fat diet (HFD, D12451 from Research Diets, Inc., New Brunswick, NJ, USA; 20% kcal protein, 35% kcal carbohydrate, 45% kcal from fat) was provided during the subsequent 3 weeks of chronic stress (18). Subordinate mice were pair fed to their baseline food intake during the first week of stress, while during the 3 weeks of HFD they were pair fed to food ingested by the control group (therefore, the normal transient hyperphagic response to palatable diet-induced shown by control mice was not prevented). Body weight and food intake were measured daily. Every day at 9:30 a.m., pair fed mice were allocated the food calculated on the basis of the amount of food that they consumed during baseline (week 1 on standard diet); during the following weeks (3 weeks on HFD), the mice were assigned the amount of food corresponding to the average amount consumed by all control mice the day before and normalized according to the subject’s respective weight.

#### Glucose tolerance tests

At the end of the stress phase, mice were subjected to an overnight fast (12 h) to then be sampled for blood glucose levels from tail bleeding at 0, 30, 60, and 120 min after intra-peritoneal injection of 0.1 cc/10g body weight of d-glucose at 10% dissolved in sterile saline solution (corresponding to 1 g/kg body weight). All blood glucose measurements were done using Accucheck Aviva glucometer (Roche Diagnostics, Indianapolis, IN, USA). The area under the curve (AUC) data for GTT were calculated according to the trapezoid rule and including all incremental area below the curve and expressed as milligram/deciliter^∗^120 min, ×10^3^.

### Terminal measures

Mice were fasted overnight before sacrifice. At autopsy, adipose fat mass was dissected and weighted. Glucose was measured as detailed above from plasma samples, while corticosterone and total ghrelin levels were measured in plasma with xMAP or ELISA commercially available kits (Bio-Rad and Millipore).

### Data analysis

Data were analyzed with Statistica (Statsoft, Inc., Tulsa, OK, USA). Repeated measures ANOVA followed by Tukey’s HSD *post hoc* tests for binary comparisons was used for meal-pattern analysis parameters, acute hyperphagia time course, and GTT; one-way ANOVA followed by Tukey’s HSD post doc was used for single time acute hyperphagia after hunger or stress, perigonadal white adipose tissue (WAT) weight, ghrelin, glucose, corticosterone levels, and GTT AUC. Repeated measures ANCOVA and Bonferroni–Holm corrected planned comparisons for binary contrasts were used for food intake and body weight time courses in the pair-feeding experiment using the respective baseline values as covariates; Significance was set at *p* < 0.05 or lower.

## Results

### Chronic subordination stress replicates key traits of binge-eating disorder

**Figure 1:**
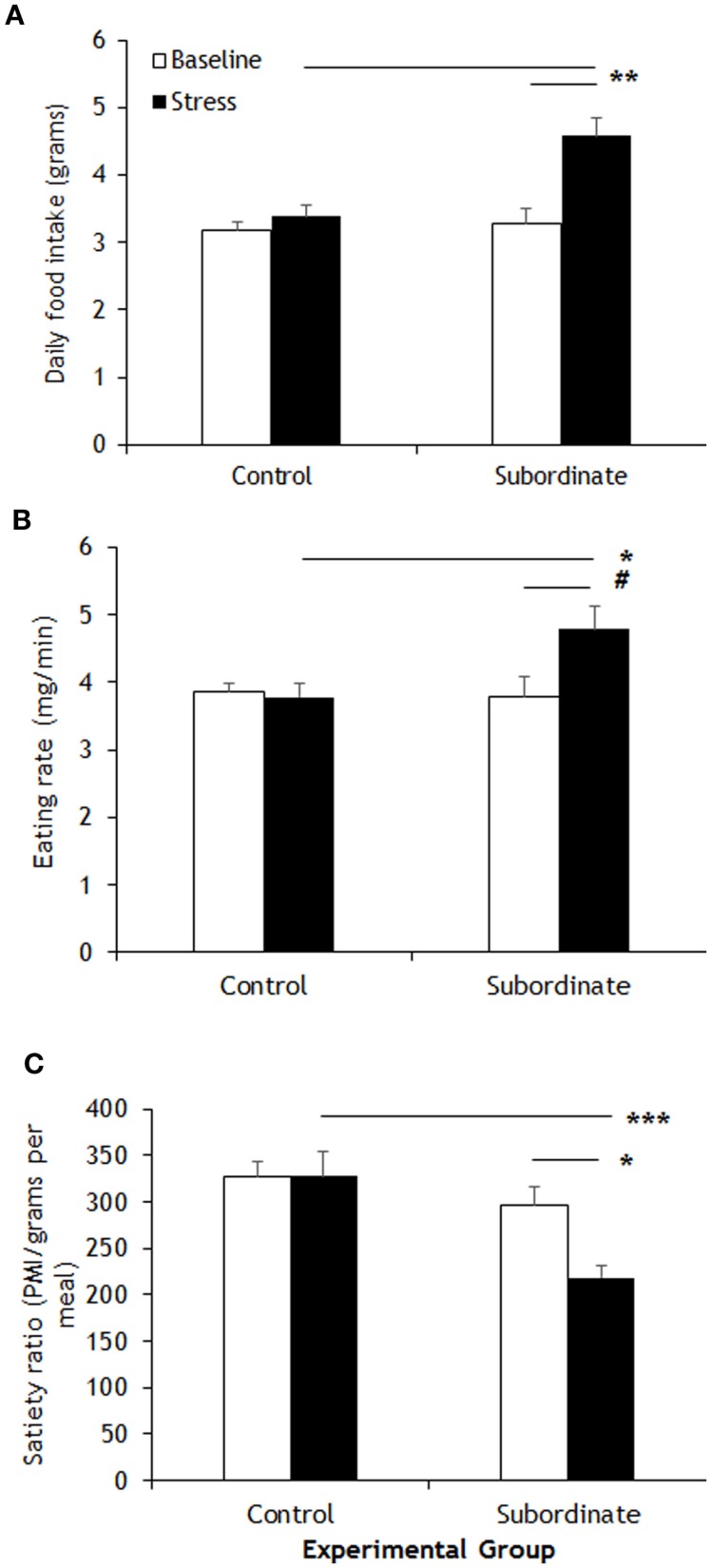
**Meal-pattern analysis highlighted increased food intake in subordinate C57BL/6J mice (A) that was associated to a faster feeding rate (B) and to a shorter satiety ratio (C) than baseline and/or controls**. Data represent group averages ± SEM. Control: *N* = 7; subordinate: *N* = 5. ^#^*p* = 0.055, *p < 0.05, **p < 0.01, ***p < 0.001.

The CSS model has proven a valuable tool to induce a positive energy balance in male mice of various strains including CD1, C57BL6/J, 129SVEV, and others (19–22, 30). Hyperphagia develops shortly after exposing the mice to subordination stress; it remains sustained throughout the stress exposure and is associated to higher body weight gain compared to controls (21). A detailed analysis of control and subordinate mice eating behavior was conducted through an automatic food monitoring system and a dedicated software generating a meal-pattern analysis. Overall, our analysis revealed a consistent CSS-induced disruption of the meal architecture, which is consistent with a BED. The overall amount of food consumed by subordinate mice was significantly greater than during baseline [*F*(1,10) = 16.50, *p* < 0.01] and compared to controls [*F*(1,10) = 8.445, *p* < 0.05] (Figure 1A). In line with derangements toward a BED-like syndrome, the eating rate was significantly increased [*F*(1,10) = 6.13, *p* < 0.05] (Figure 1B), while the satiety ratio was significantly decreased by CSS [interaction *F*(1,10) = 8.77, *p* < 0.05. Figure 1C). Conversely, meal frequency, duration, and size, as well as post-meal interval and total meal time were not different between subordinate and control group (Table 1), suggesting that only specific components of satiation were targeted by stress. The lack of changes in other meal parameters could be a compensatory response or imply that long term control of feeding remained intact.

**Table 1:** **Meal-pattern parameters that did not correspond to any significant differences between groups**.

	Control		Stress	
Parameter	Baseline	Stress phase	Baseline	Stress phase
Meal frequency (number/day)	14.29 : 0.49	16.25 : 1.42	15.48 : 1.77	18.20 : 1.59
Meal duration (min/day)	23.99 : 2.18	21.89 : 2.29	30.84 : 3.28	23.64 : 3.42
Post-meal interval (min)	72.61 : 2.97	68.94 : 5.62	64.49 : 7.48	56.01 : 4.38
Meal size (g/meal)	0.22 : 0.01	0.22 : 0.02	0.22 : 0.01	0.26 : 0.02
Total meal time (min/day)	365.78 : 22.83	341.43 : 22.52	454.280 : 5.67	411.58 : 24.16

*Data represent group averages ± SEM. Control *N* = 7; subordinate *N* = 5*.

### CSS enhances the susceptibility to acute hunger- and stress-induces binge-eating episode

**Figure 2:**
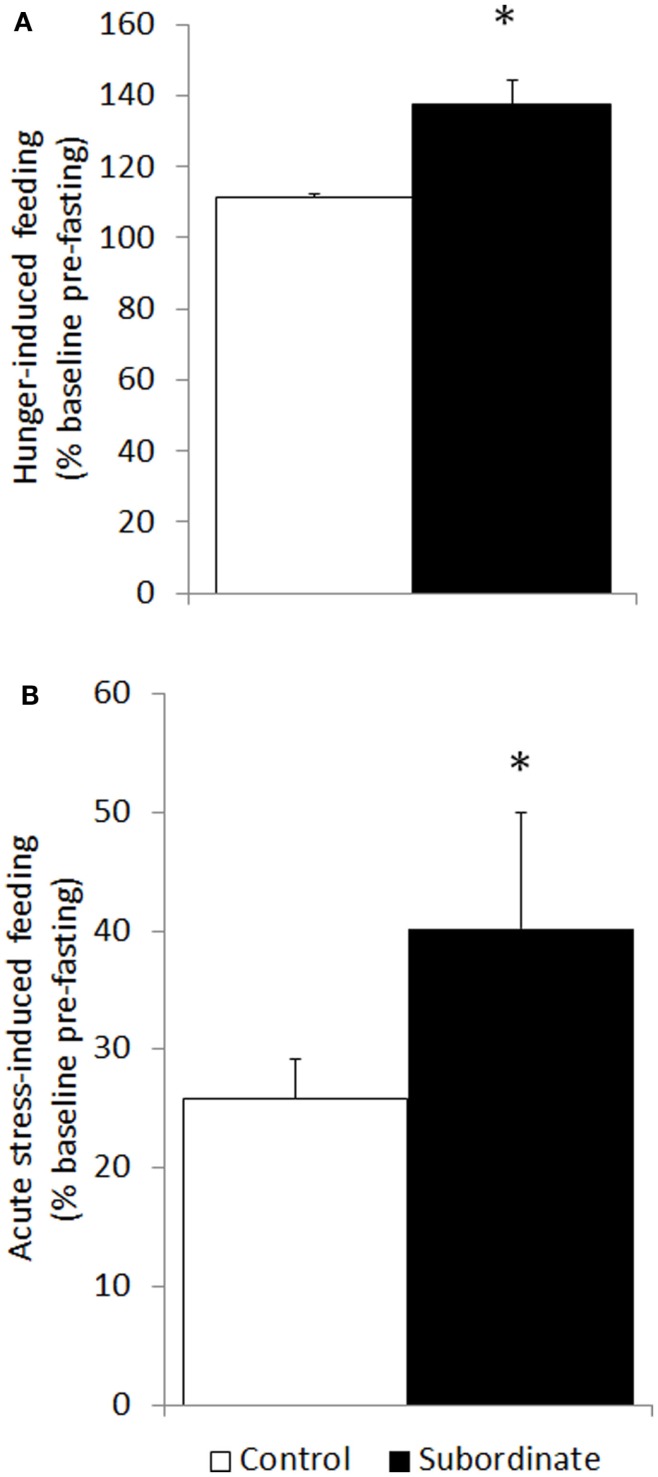
**Hyperphagia was further exacerbated in subordinate CD1 mice in response to acute stress (social defeat for 10 min) in the subsequent 6 h (A) as well as to overnight fasting followed by refeeding (B)**. Data represent group averages ± SEM. **(A)** Control: *N* = 15; subordinate: *N* = 13. **(B)** Control: *N* = 15; subordinate: *N* = 10. *p < 0.05, ***p < 0.001.

**Figure 3:**
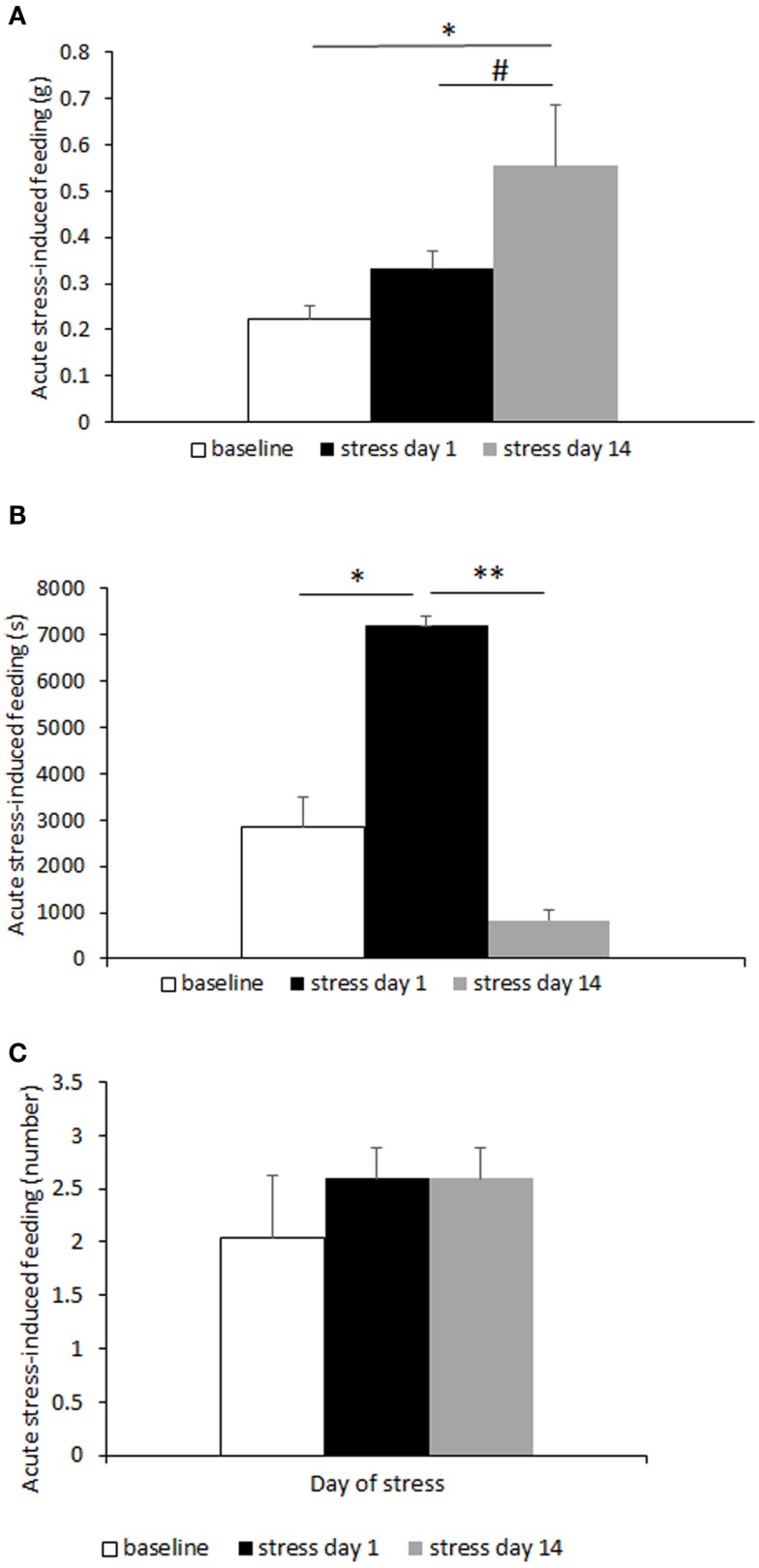
**Meal-pattern and time-course analysis of acute stress-induced hyperphagia in C57BL/6J mice**. Meal intake was increased over time in subordinate **(A)**, while meal duration **(B)** was initially increased to later on diminish in correspondence with a meal frequency that remained stable over time **(C)**. Data represent group averages ± SEM. Control: *N* = 7; subordinate: *N* = 5. ^#^*p* = 0.055, *p < 0.05, **p < 0.01.

Increased hunger- and stress-induced hyperphagia are classically accepted as behavioral markers of BED (10, 31). Hunger-induced hyperphagia was here assessed in both CD1 and C57BL6/J mice in a fasting–refeeding protocol. Subordinate CD1 mice ingested a significantly larger amount of food compared to controls [*F*(1,23) = 21.88, *p* < 0.001] (Figure 2A). Similarly, in the 6 h after a single episode of social defeat on the 14th day of the CSS, subordinate mice showed increased food intake [*F*(1,25) = 5.32, *p* < 0.05] (Figure 2B). Furthermore, we repeated and extended this observation by using the automated analysis of food intake system. During 6 h immediately following the social defeat, meal intake was significantly increased in subordinate C57BL6/J mice [*F*(2,8) = 7.12, *p* < 0.05] (Figure 3A) with a maximal intake occurring after 14 days of stress (*p* < 0.05). Interestingly, meal duration showed a biphasic effect [*F*(2,8) = 16.23, *p* < 0.01] (Figure 3B) being increased on the first day of stress compared to baseline (*p* < 0.05) and being reduced at day 14 (*p* < 0.01). Nevertheless, the number of meals exhibited by subordinate mice throughout the stress exposure did not change over time (Figure 3C).

### Stress-induced hyperphagia is required for the development of obesity in our animal model

**Figure 4:**
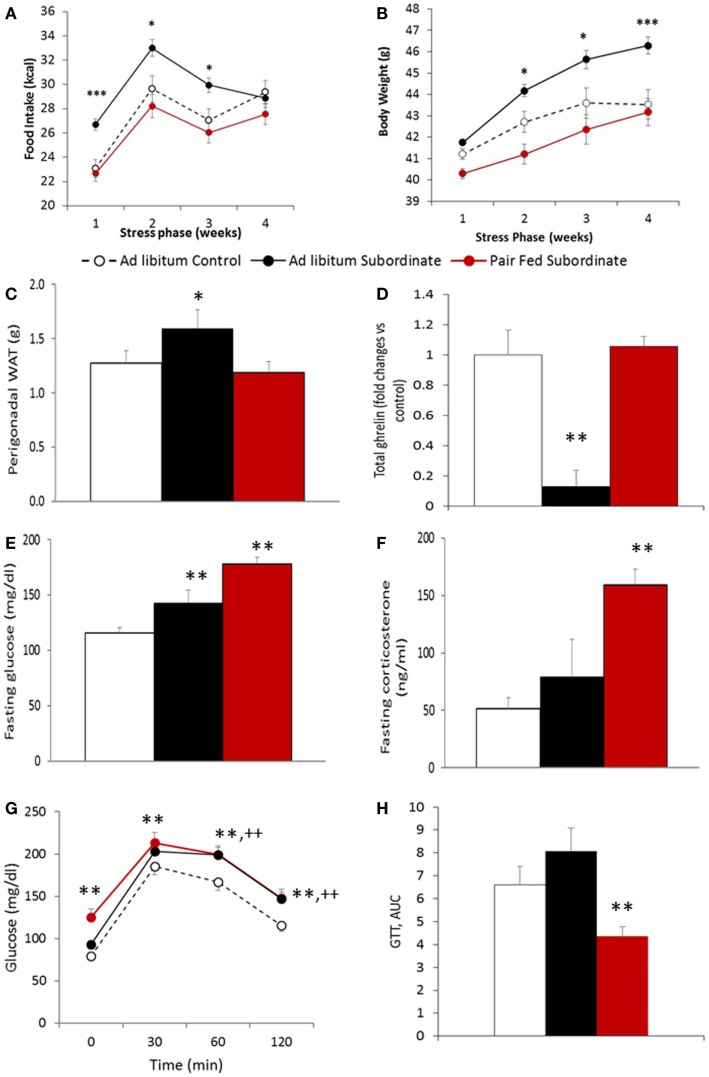
**Pair-feeding subordinate mice prevents stress-induced vulnerability to diet-induced obesity**. **(A)** Food intake, data are presented as least square means ± SEM; the covariate is the baseline food intake, average = 22.5 kcal [*F*(1,49) = 22.24, *p* < 0.01; *ad libitum* fed control: *N* = 12; *ad libitum* fed subordinate: *N* = 28; pair fed subordinate: *N* = 13]; **(B)** body weight gain, data are presented as least square means ± SEM; the covariate is the baseline body weight, average = 41.2 g [*F*(1,55) = 198.37, *p* < 0.001; *ad libitum* fed control: *N* = 22; *ad libitum* fed subordinate: *N* = 34; pair fed subordinate: *N* = 13]; **(C)** perigonadal white adipose tissue (WAT) [*ad libitum* fed control: *N* = 12; *ad libitum* fed subordinate: *N* = 26; pair fed subordinate: *N* = 12]; **(D)** total ghrelin [*ad libitum* fed control: *N* = 5; *ad libitum* fed subordinate: *N* = 6; pair fed subordinate: *N* = 8]; **(E)** glucose [*ad libitum* fed control: *N* = 21; *ad libitum* fed subordinate: *N* = 26; pair fed subordinate: *N* = 9]; **(F)** corticosterone [*ad libitum* fed control: *N* = 17; *ad libitum* fed subordinate: *N* = 23; pair fed subordinate: *N* = 8]; **(G,H)** glucose tolerance test [*ad libitum* fed control: *N* = 16; *ad libitum* fed subordinate: *N* = 19; pair fed subordinate: *N* = 13]. **(D–H)** Data represent group averages ± SEM, *p < 0.05, **p < 0.01, ***p < 0.001 vs. control. In **(G)**, ** refer to binary comparisons between pair fed subordinate and control mice, while ++ refer to binary comparisons between *ad libitum* fed subordinate and control mice.

After having established that CSS leads to features of BED, we directly tested the hypothesis that hyperphagia is required for the development of weight gain, fat mass increase, and glucose intolerance in CD1 animals fed an HFD as previously established (21). To test this hypothesis, hyperphagia was prevented by pair-feeding subordinate mice an amount of food similar to pre-stress food intake or control mice (Figure 4A). Importantly, pair feeding prevented CSS-induced weight gain (Figure 4B) and increased adiposity (Figure 4C) shown by *ad libitum* fed mice [food intake: *F*(2,49) = 11.62, *p* < 0.001; body weight: *F*(2,55) = 13.44, *p* < 0.001; pWAT *F*(1,56) = 3.4577, *p* < 0.05]. Interestingly, total ghrelin was significantly decreased by CSS in *ad libitum* fed mice and normalized by pair feeding [*F*(2,26) = 7.49, *p* < 0.001] (Figure 4D).

Despite pair feeding preventing the development of obesity, it did not prevent the development of hyperglycemia while it improved glucose tolerance. Indeed, pair-fed subordinate mice showed higher fasting hyperglycemia than both control and *ad libitum* fed subordinate mice [*F*(2,53) = 16.072, *p* < 0.001] (Figure 4E). This result can be explained by the very high level of fasting corticosterone observed in pair-fed stressed animals [*F*(2,45) = 7.6222, *p* < 0.01] (Figure 4F). Additionally, despite pair-fed CSS mice having a significant hyperglycemia compared to *ad libitum* fed CSS mice, the glucose level of the two stressed groups was indistinguishable after glucose injection [*F*(2,44) = 8.61, *p* < 0.001] (Figure 4G), which resulted in a significantly lower AUC for the pair-fed CSS compared to the *ad libitum* CSS group [*F*(2,42) = 53.58E+06, *p* < 0.01] (Figure 4H), overall suggestive of improved glucose tolerance in the pair fed CSS compared to *ad libitum* fed CSS mice.

## Discussion

In the present study, we demonstrated that the outcome of CSS model resembles many features of human BED. Spontaneous hyperphagia is one of the most robust phenotypes observed in mice exposed to the chronic psychosocial stress model [present work; (17–20, 22)] as well as in similar animal models of social stress (32, 33). Subordinate mice spontaneously developed vigorous hyperphagia (~30–40% than baseline) characterized by early onset (1–2 days) and lack of habituation (i.e., up to 4 weeks). Importantly, we previously showed that hyperphagia develops in absence of increased energy expenditure (21, 23) and in presence of depression of locomotor activity (18–20, 30) thus excluding a compensatory hyperphagia. In light of these evidences and of the behavioral characterization of the subordinate mice as a model of

depression-like disorder (15, 17, 19, 20), we hypothesized that the observed hyperphagia can be considered as a specific eating disorder phenotype having a strong validity for modeling human binge eating associated with obesity (1–5, 34, 35). Accordingly, we tested in our model the specific criteria used for the diagnosis of BED in human (Table 2). Specifically, we explored the microstructure of spontaneous daily feeding behavior as well as the structure of the feeding response in response to acute episodes of hunger and stress (36, 37). It was demonstrated that the overall hyperphagia is indeed the result of an increased amount of ingested food, which subordinate mice consume at higher rate and with reduced satiety ratio than control mice. The observed decreased satiety ratio (Figure 1C) point toward a reduced effectiveness of food ingestion to induce satiety (38), while the increased rate of food intake (Figure 1B) would reflect feeding while eating in a perceived stressful situation as feeding rate is known to be augmented by stress (39) and in social feeding in subordinate individuals (40).

**Table 2:** **A comparison between symptoms of binge-eating disorder (DSM-V) and effects seen in subordinate mice under chronic subordination stress**.

Diagnostic criteria for the diagnosis of binge-eating disorder (DSM-V)	Effect in our animal model
A. Recurrent episodes of binge eating characterized by both of the following	
1. Eating, in a discrete period of time, an amount of food that is larger than normal.	1. Increased eating rate (g of food/min). Acute stress/hunger-induced hyperphagia
2. A sense of lack of control over eating during the episode.	2. Decreased satiety ratio

B. The binge-eating episodes are associated with three (or more) of the following	
1. Eating much more rapidly than normal	1. Shorter meals revealed by meal pattern
2. Eating until feeling uncomfortably full	2. Not applicable or not available
3. Eating large amounts of food when not feeling physically hungry	3. Short interval between feeding bouts revealed by meal pattern analysis. High acute stress-induced feeding during the light phase
4. Eating alone because of feeling embarrassed by how much one is eating	4. Not applicable or not available
5. Feeling disgusted with oneself, depressed, or very guilty after overeating	5. Development of depression-like disorder

C. Marked distress regarding binge eating	C. Biomarkers of stress and acute stress-induced hyperphagia

D. The binge eating occurs at least once a week for 3 months	D. Recurrent episodes of binge eating during the entire stress phase

E. The binge eating is not associated with the recurrent use of inappropriate compensatory behavior (such as purging).	E. Not applicable or not available

Interestingly, the sustained hyperphagia induced in subordinate mice could be further exacerbated acutely in response to either hunger or social defeat, both of which triggered increased amount of food intake over discrete periods of time. The feeding microstructure analyzed in the immediate sequelae of individual social defeat showed that eating behavior of subordinate mice lasted longer and was composed of a higher number of meals on the day on the first social defeat episode in the CSS protocol. The alteration of meal structure due to subordination stress changed over time. When assessed at day 14 in the subordination stress phase, the feeding activity of subordinate mice had evolved into an eating paroxysm, as their behavior consisted of a maximal amount of food consumed over much shorter meal times. Overall, these results allow to validate the CSS accordingly to the defining criteria of a BED model (9) (Table 2): (1) the behavior occurs repeatedly over an extended period of time; (2) binging animals consume more food in brief, discrete, periods of time than controls do under similar circumstances. (3) if compensatory behavior is present, it should be initiated by the animal rather than imposed by the investigator. It is also worth to point out that this behavior was observed during a phase of the day corresponding to the natural mice resting phase (6 h following dominant/intruder interaction, with social defeat being carried out between 8:30 a.m. and 9:30 a.m.). Interestingly, in obese populations, BED has been linked with night-eating syndrome (41–44) and nocturnal snacking (45, 46).

Another important feature of our model is that binge eating develops spontaneously and is associated with derangements toward obesity and the metabolic syndrome [present data and Ref. (21)]. Because obesity is a major risk factor for the development of the metabolic syndrome and type-2 diabetes (47–50), a food restriction regimen promoting weight loss should normalize the metabolic syndrome as well (51). Preventing hyperphagia using a pair-feeding protocol in subordinate mice completely abrogated body weight gain and visceral adiposity compared to subordinate mice that were fed *ad libitum*. This result has clear translational implications toward therapeutic interventions (52): offering the potential of treating obesity in a faster way (drugs are often effective after several weeks of treatment); treating obesity with non-pharmacological interventions effective for binge eating. Some of the involved peripheral mechanisms were here investigated, while the microstructural analysis of feeding could help pinpoint whether changes in meal parameter are due to altered negative feedback, which is mainly vagally mediated vs. altered palatability or motivation, which is mainly central nervous system in origin (53). Nevertheless, a lot remains to be established particularly at the central level. Previous studies [e.g., Ref. (13, 17, 19, 54–59)] suggest that central peptidergic and monoaminergic pathways are likely candidates as the molecular substrate of the stress-associated BED-like syndrome. Food intake regulation indeed impinges on the integration of homeostatic and hedonic brain circuits, which mediate the drive for food intake depending on energy store levels and on rewarding properties of foods (60). Interestingly, hypothalamic levels of orexigenic peptides, such as NPY and AgRP, have indeed been found in the hypothalamus of hyperphagic subordinate mice (22). Despite progress have been made, future studies will need to be performed to identify the central pathways regulating subordination stress-induced BED-like syndrome.

In spite of the protective effects of preventing hyperphagia on obesity, pair feeding improved glucose tolerance but did not normalize fasting hyperglycemia (21). It is important to point out that the basal hyperglycemia of pair-fed subordinate mice was obtained in response to fasting and could indicate, in line with the results of the meal pattern analysis, more severe hunger-stress sensitivity, as highlighted by the parallel heightened corticosterone. Ghrelin is a hunger hormone previously associated with stress (22, 61–64) and decreased in the plasma of obese patients (65, 66). Fasting plasma ghrelin (67, 68) was decreased in subordinate *ad libitum* fed mice when compared to controls a finding in line with human studies, but it was normalized in subordinate pair fed mice. Conversely, other studies showed that the active form of ghrelin, i.e., acyl-ghrelin, was increased in non-fasted subordinate mice in presence of increased (22, 69) or decreased (70, 71) weight gain. Although recent data suggest that also des-acyl-ghrelin might exert a biological activity, which appears to be independent from the GHSR-1a (72, 73), a limitation of the present study is that total and not acyl-ghrelin was assessed. Accordingly, future studies are required to evaluate how CSS affects the active vs. inactive circulating level of this hormone if the levels are affected by fasting in similar models and subordinate mice also manifest a postprandial suppression of acyl-ghrelin levels as seen in patients.

In conclusion, we propose here a novel model of BED, which is characterized by spontaneous stress-associated binging episodes and is associated with the development of obesity and glucose intolerance. We also showed that preventing hyperphagia limits the development of diet-induced obesity. Our social subordination model convincingly provides a tool to access the mechanisms that amount to the regulation of eating behaviors both in health and pathological states, ultimately trying to deliver therapeutic solutions to human patients (74). A limitation of the CSS model is that it can only be applied to males. Indeed, laboratory bred female mice neither exhibit territorial aggression nor they manifest a robust social hierarchy (75). Nevertheless, models of BED have been validated and made available specifically in female mice (13). This creates the opportunity for an integrated comparative approach based on highly valid models for male and female mice and it holds the promise to highlight important aspects related to gender differences in BED observed in humans (76).

## Conflict of Interest Statement

The authors declare that the research was conducted in the absence of any commercial or financial relationships that could be construed as a potential conflict of interest.
